# Metabolic Syndrome as a Risk Factor for Elevated Intraocular Pressure

**DOI:** 10.12669/pjms.303.4514

**Published:** 2014

**Authors:** Nedime Sahinoglu-Keskek, Sakir Ozgur Keskek, Selim Cevher, Sinan Kirim, Asim Kayiklik, Gulay Ortoglu, Tayyibe Saler

**Affiliations:** 1 Nedime Sahinoglu-Keskek, MD, Department of Ophthalmology, Numune Training and Research Hospital, Adana, Turkey.; 2 Sakir Ozgur Keskek, MD, Department of Internal Medicine, Numune Training and Research Hospital, Adana, Turkey.; 3 Selim Cevher, MD, Department of Ophthalmology, Numune Training and Research Hospital, Adana, Turkey.; 4 Sinan Kirim, MD, Department of Internal Medicine, Numune Training and Research Hospital, Adana, Turkey.; 5 Asim Kayiklik, MD, Department of Ophthalmology, Numune Training and Research Hospital, Adana, Turkey.; 6 Gulay Ortoglu, MD, Department of Internal Medicine, Numune Training and Research Hospital, Adana, Turkey.; 7 Tayyibe Saler, Assistant Professor, Department of Internal Medicine, Numune Training and Research Hospital, Adana, Turkey.

**Keywords:** Intraocular pressure, Central corneal thickness, Metabolic syndrome, Glaucoma

## Abstract

***Objective:*** The aim of this study was to investigate the association between intraocular pressure and metabolic syndrome by comparing central corneal thicknesses.

***Methods:*** One hundred sixty-two subjects were enrolled in this cross-sectional study, with 89 subjects in a metabolic syndrome group and 73 subjects in a control group. Ophthalmological examinations, including intraocular pressure and central corneal thickness measurements, were performed on each subject. Serum fasting glucose, triglyceride and HDL cholesterol levels were measured, and waist circumference, systolic and diastolic blood pressure of all patients were recorded.

***Results:*** Participants with metabolic syndrome had a significantly higher intraocular pressure than those without metabolic syndrome (p = 0.008), and there was no statistically significant difference between the central corneal thicknesses of the two groups (p = 0.553). Most of the metabolic syndrome components were associated with higher intraocular pressure (p < 0.05).

***Conclusions:*** There is a relationship between metabolic syndrome and intraocular pressure, but no association between metabolic syndrome and central corneal thicknesses. Intraocular pressure is affected by central corneal thicknesses, and intraocular pressure is used to correct according to the central corneal thicknesses measurement. To our knowledge, this is the first study that determines the positive relationship between metabolic syndrome and intraocular pressure by comparing the central corneal thicknesses of the groups.

## INTRODUCTION

Glaucoma is a significant cause of irreversible blindness worldwide^1^^,^^2^, and intraocular pressure (IOP) is a modifiable risk factor for primary open angle glaucoma (POAG).^3^ Metabolic syndrome is a cluster of metabolic abnormalities that includes hypertriglyceridaemia, low levels of high-density lipoprotein cholesterol, obesity, hypertension and hyperglycaemia. Diabetes, hypertension and obesity have been found to be associated with elevated IOPs^4^^-^^12^, and according to previous studies, elevated IOP is an expected condition in metabolic disturbances that are associated with the components of metabolic syndrome (MS).^13^^,^^14^

The correlation between central corneal thickness (CCT) and IOP is well known. Eyes with greater mean CCTs tend to have higher IOPs.^15^^,^^16^ In our study, we compared the CCTs of the patients to determine the relationship between metabolic syndrome and IOP. 

Metabolic syndrome is a common condition in Turkey. The purpose of this study was to examine the relationship between metabolic syndrome and IOP, and to compare the IOP levels of those patients with those of normal subjects.

## METHODS

***Study participants: ***In this cross-sectional study, we analysed 162 subjects in two groups: one group of 89 metabolic syndrome patients and a control group of 73 healthy subjects. Approval for the study protocol was granted by the Adana Numune Training and Research Hospital’s institutional review board, and the study was conducted in accordance with the Declaration of Helsinki. The medical histories and conditions of the participants were carefully ascertained by physicians, including ophthalmologists. Written informed consent was obtained from all participants before enrolment. Participants with past histories of glaucoma, surgery, use of steroid drugs or diabetic retinopathy were excluded.

***Data collection: ***The collection of the data for this study included the following tests: ophthalmological examination, blood chemistry, waist circumference and blood pressure. Blood pressure was measured using a mercury sphygmomanometer, and three separate readings at one minute intervals were taken by trained nurses. A venous blood sample was collected in the morning after overnight fasting. Fasting glucose, triglyceride and high-density lipoprotein (HDL) levels were analysed with a Roche C-501 (Tokyo, Japan) by using the hexokinase method (glucose) and homogeneous colorimetric enzyme test (triglyceride and HDL). Metabolic syndrome was defined according to the National Cholesterol Education Program third Adult Treatment Panel guidelines (NCEP ATP III).^13^

The components of metabolic syndrome included: waist circumference > 102 cm in men or > 88 cm in women, triglyceride ≥ 150 mg per 100 ml, HDL < 40 mg per 100 ml in men or < 50 mg per 100 ml in women, blood pressure ≥ 130/85 mmHg and fasting glucose ≥ 100 mg per 100 ml. Metabolic syndrome was defined as having at least three of the five components.^13^

Ophthalmological examinations were performed between 8:00 am and 11:00 am, and included the best-corrected visual acuity, refraction, IOP measurement by non-contact tonometry (Canon Auto Tonometer TX-F, Canon, USA) and dilated fundus examination. A trained nurse took three consecutive measurements for each eye, and the average IOP of the right eye was used for analysis. Ocular hypertension was defined as an IOP ≥ 21 mmHg in the right eye. Five CCT measurements were obtained from each eye with a Sonomed ultrasound pachymeter (PacScan 300P, California, USA), and the median reading was taken.

***Statistical methods: ***MedCalc 12.0 software (MedCalc, Turkey) was used for the statistical analysis, and data were reported as the mean ± sd. Chi square and Kolmogorov-Smirnov tests were used to compare the categorical measurements between the groups, and to show the normal distribution of the quantitative measurements, respectively. The t- test or Mann-Whitney U test were used for the comparison of the quantitative measurements between the two groups. The Pearson correlation coefficients (r) were used in order to analyse the degree of association between the two variables (with P-value and 95% CI for r). The Log transformation was used for variables that were not normally distributed, and the level of statistical significance was considered to be 0.05 in all tests.

## RESULTS

The characteristics of the study subjects are summarized in Table-I. The mean age was 49.04 years (±10.68) for the metabolic syndrome group, and 48.10 years (±9.35) for the healthy control group. There were 37 men and 52 women in the metabolic syndrome group, and 35 men and 38 women in the control group. There was no statistical difference in the ages and gender distributions of the two groups (p values = 0.24 and 0.81, respectively). 

The mean IOP was 14.32 (±3.26) mmHg in the participants without metabolic syndrome, and 16.25 (±3.80) mmHg in those with metabolic syndrome. The subjects with metabolic syndrome had significantly higher IOP levels than those without metabolic syndrome. The prevalence of ocular hypertension was 13.48 and 4.10% in the participants with and without metabolic syndrome, respectively. An analysis of the components of metabolic syndrome showed that the participants with high blood pressure, high fasting glucose, high triglycerides and abdominal obesity had significantly higher IOP levels, when compared to the subjects without these risk factors (p < 0.05, respectively) (Table-II). No correlation was found between the low HDL levels and high intraocular pressure (Table-III). When each component of the metabolic syndrome was analysed, fasting glucose level, triglyceride level, systolic and diastolic blood pressure and waist circumference were positively associated with IOP (Table-III).

The mean values of CCT were 540.04 (±35.65) and 536.78 (±33.81) µm in the patients with metabolic syndrome and the control group, respectively. There was no significant difference in the CCT between the two groups (p = 0.553) (Table-IV). None of the components of metabolic syndrome were found to be associated with central corneal thickness (p > 0.05) (Table-V).

**Table-I:** Clinical characteristics of the study subjects according to the diagnosis of metabolic syndrome

	*n*	*With metabolic syndrome*	*Without metabolic syndrome*	*p-values*	*Reference values*
Age (years)	162	49.0±10.6	48.1±9.3	0.240	
Male (%)	75	41.6	52.1	0.810	
Waist circumference (cm)	162	117.4±18.1	83.2±9.6	<0.001	Male<102 Female<88
Systolic blood pressure (mmHg)	162	145.50±24.94	107.0±11.83	<0.001	<130
Diastolic blood pressure (mmHg)	162	86.8±15.0	66.3±9.6	<0.001	≤85
Fasting glucose (mg/dl)	162	150.3±68.6	89.9±8.2	<0.001	<110
HDL (mg/dl)	162	42.9±8.4	52.5±12.2	<0.001	Male>40 Female>50
Triglycerides (mg/dl)	162	204.7±88.3	104.0±53.9	<0.001	≤150
Intraocular pressure (mmHg)	162	16.2±3.8	14.3±3.2	0.008	≥21
Ocular hypertension (%)	162	14.6	2.7	0.035	

**Table-II:** Comparison of the mean values of intraocular pressure according to each component of metabolic syndrome

*Component of metabolic syndrome*	*n*	*Mean IOP(±SD)*	*p-value*
Abdominal obesity			
No (male) Yes (male) No (female) Yes (female)	41 34 22 65	14.09±3.54 16.26±3.77 14.14±3.41 15.97±3.68	0.012 0.043
High fasting glucose			
No Yes	83 79	14.55±3.20 16.26±3.96	0.0029
Low HDL			
No (male) Yes (male) No (female) Yes (female)	59 16 29 58	15.33±3.87 14.12±3.38 16.24±3.42 15.36±3.64	0.258 0.285
High triglycerides			
No Yes	88 74	14.78±3.69 16.10±3.56	0.019
High systolic blood pressure No Yes	90 72	14.48±3.12 16.67±3.96	0.0004
High diastolic blood pressure			
No Yes	106 56	14.81±3.26 16.48±4.19	<0.0057
Metabolic syndrome			
No Yes	73 89	14.32±3.26 16.25±3.80	0.0008

The components of metabolic syndrome were: abdominal obesity, waist circumference > 102 cm in men or > 88 cm in women; high triglyceride, ≥ 150 mg per 100 ml; low HDL, < 40 mg per 100 ml in men or < 50 mg per 100 ml in women; high blood pressure, ≥ 130 for systolic and ≥ 85 mmHg for diastolic pressure; and high fasting glucose, ≥ 100 mg per 100 ml

**Table-III:** Association of IOP with metabolic parameters

Metabolic parameters		IOP
Waist circumference	r p	0.229 0.003
Systolic blood pressure	r p	0.257 0.0009
Diastolic blood pressure	r p	0.210 0.006
Fasting glucose	r p	0.220 0.004
HDL	r p	0.023 0.769
Triglycerides	r p	0.224 0.004

**Table-IV:** Comparison of the mean values of CCT according to each component of metabolic syndrome

*Component of metabolic syndrome*	*n*	*Mean CCT(±SD)*	*p-value*
Abdominal obesity			
No (male) Yes (male)	41 34	538.7±35.5 538.3±30.8	0.964
No (female) Yes (female)	22 65	540.2±42.7 538.0±34.5	0.813
High fasting glucose			
No Yes	83 79	535.7±32.6 541.5±36.8	0.292
Low HDL			
No (male) Yes (male) No (female) Yes (female)	59 16 29 58	538.0±34.2 540.3±27.8 536.0±36.3 539.9±36.8	0.800 0.643
High triglycerides			
No Yes	88 74	539.2±33.3 537.7±36.5	0.784
High systolic blood pressure			
No Yes	90 72	536.5±32.8 541.1±37.1	0.407
High diastolic blood pressure			
No Yes	106 56	539.8±34.7 536.2±35.0	0.538
Metabolic syndrome			
No Yes	73 89	536.7±33.8 540.4±35.6	0.553

**Table-V:** The association of CCT with the metabolic parameters

*Metabolic parameters*		*CCT*
Waist circumference	r p	0.005 0.940
Systolic blood pressure	r p	0.097 0.217
Diastolic blood pressure	r p	0.050 0.523
Fasting glucose	r p	0.025 0.744
HDL	r p	-0.059 0.449
Triglycerides	r p	-0.015 0.846

## DISCUSSION

We found that metabolic syndrome and its components were associated with higher intraocular pressure, and it has been previously reported that subjects with metabolic syndrome had higher IOPs.^13^^,^^14^^,^^17^ This study also showed that four of the five components (fasting plasma glucose, blood pressure, waist circumference and triglyceride) were associated with higher IOPs.

Previous studies have reported positive associations between the body mass index and IOP.^18^^-^^21^ Higher IOPs in obesity are likely due to excess intraorbital fat tissue, an increase in the viscosity of the blood, and an increase in the episcleral venous pressure, which, consequently, decrease the outflow facility.^5^^,^^12^^,^^19^

The current study found significant relationships in both the systolic and diastolic blood pressures with IOP. Lin et al., investigated the relative importance of each component of metabolic syndrome in specific age and gender groups in their study^22^, and found that elevated IOP was associated with different metabolic variables according to the patient groups: BMI for young adults, fasting blood sugar for older adults, diastolic blood pressure for men and systolic blood pressure for most participants. They also reported blood pressure to be the strongest predictor of elevated IOP.

Other studies in the literature suggested that the causes of high IOP in systemic hypertension are the excessive production of aqueous humour, and the increase in the episcleral venous and ciliary arterial pressures.^21^^,^^23^ Additionally, increased sympathetic tone and serum corticosteroids were found to be responsible for high IOPs in patients having high blood pressure.^24^

There was a correlation between high-ocular tension and hyperglycaemia in our study; however, the mechanism behind how hyperglycaemia affects IOP is unclear. The osmotic gradient induced by elevated blood glucose, with a consequent fluid shift into the intraocular space, and autonomic dysfunction having been proposed to be the factors explaining the association.^25^^,^^26^

Previous studies have shown the positive relationship between IOP and total cholesterol and triglycerides.^5^^,^^27^ Similarly, there is a positive correlation between high IOP and high triglycerides in our study. Possible reasons for this are that hypertriglyceridemia is commonly associated with obesity.

We suspect that a common background exists between high ocular tension and metabolic syndrome, and some potential pathophysiological mechanisms about metabolic syndrome and IOP have been revealed by recent research. Obesity, hypertension and insulin resistance cause sympathetic hyperactivation^28^^,^^29^, and IOP increases with the stimulation of sympathetic activation.^30^ It has also been reported that β-adrenergic receptor polymorphism is associated with obesity, insulin resistance, IOP and POAG.^29^^,^^31^ It has been shown that IOP is affected by the endogenous rhythm, where IOP rises in the winter and is negatively related to body temperature.^5^^,^^27^^,^^32^

The endocannabinoid pathway is defined as a pathway which controls energy homoeostasis and appetite^33^, and abdominal obesity, dyslipidaemia, hyperglycaemia and hepatic lipogenesis exist due to endocannabinoid overactivity.^33^ Endocannabinoid receptors are also present in various eye tissues, including the trabecular meshwork, ciliary body, corneal epithelium and the canal of Schlemm.^34^ It has been shown in rodents that when administered intravenously or topically, endocannabinoids regulate aqueous outflow in the trabecular meshwork and influence IOP.^34^ Aquaporins are a family of membrane proteins transporting water and small molecules, such as glycerol. They are expressed in the corneal endothelium, trabecular meshwork, ciliary epithelium, choroid plexus, adipose tissue, pancreas and liver.^35^ Aquaporins regulate the IOP by increasing aqueous fluid secretions across the ciliary epithelium.^35^

Park et al. studied the association between IOP and metabolic syndrome according to menopause status in nonglaucomatous Korean women.^36^ They found that elevated IOP was associated with metabolic syndrome in postmenopausal women, but not in premenopausal women. They reported that oestrogen could directly influence target tissues in the eye, such as the ciliary epithelia, trabecular meshwork, subepithelial vascular plexus and episcleral venous plexus, as well as act on the inflow of the aqueous humour.

Central corneal thickness is an important factor effecting intraocular pressure, and extremes in the CCT can lead to errors in IOP measurement. To our knowledge, this is the first study performed to investigate the correlation between metabolic syndrome and intraocular pressure changes by analysing the CCTs of patients. There was no statistical significance found between the CCTs of the metabolic syndrome group and the control group. However, it can be concluded that there is a real relationship between metabolic syndrome and elevated intraocular pressure.

In this study, there was no association between the CCT and any of the metabolic syndrome components. Some studies in the literature highlighted that CCT was greater in individuals with diabetes and metabolic syndrome.^17^^,^^37^ The reason for this association is unclear, but there is the hypothesis that these conditions alter corneal endothelial physiology, leading to an increase in CCT.^38^^,^^39^

The cross-sectional design of this study limited its ability to establish a causal relationship between metabolic syndrome and IOP. Cohort studies or clinical trials will help us to determine the exact causal relationships between the identified risk factors and IOP. 

Sedentary lifestyles cause increases in the prevalence of the metabolic syndrome, and lifestyle intervention can provide an amelioration of metabolic syndrome and decrease IOP. However, further longitudinal studies are required to clarify the relationship between insulin resistance and IOP.
